# Prospective Validation of the Laboratory Risk Indicator for Necrotizing Fasciitis (LRINEC) Score for Necrotizing Fasciitis of the Extremities

**DOI:** 10.1371/journal.pone.0227748

**Published:** 2020-01-24

**Authors:** Cheng-Ting Hsiao, Chia-Peng Chang, Tsung-Yu Huang, Yi-Chuan Chen, Wen-Chih Fann

**Affiliations:** 1 Department of Emergency Medicine, Chang Gung Memorial Hospital, Chiayi, Taiwan; 2 School of Medicine, Chang Gung University, Taoyuan, Taiwan; 3 Division of Infectious Diseases, Department of Internal Medical, Chang Gung Memorial Hospital, Chiayi, Taiwan; 4 Department of Nursing, Chang Gung University of Science and Technology Chiayi Campus, Chiayi, Taiwan; University of Porto Faculty of Medicine, PORTUGAL

## Abstract

**Objectives:**

The Laboratory Risk Indicator for Necrotizing Fasciitis score was developed as a clinical decision tool for distinguishing necrotizing fasciitis from other soft tissue infections. We prospectively evaluated the performance of the Laboratory Risk Indicator for Necrotizing Fasciitis score for the diagnosis of patients with necrotizing fasciitis in the extremities.

**Methods:**

We conducted a prospective and observational cohort study of emergency department patients with necrotizing fasciitis or severe cellulitis in the extremities between April 2015 and December 2016. The Laboratory Risk Indicator for Necrotizing Fasciitis score was calculated for every enrolled patient. The sensitivity, specificity, positive predictive value, and negative predictive value of cut-off scores of 6 and 8 were evaluated. The accuracy of the Laboratory Risk Indicator for Necrotizing Fasciitis score was expressed as the area under the receiver operating characteristic curve.

**Results:**

A total of 106 patients with necrotizing fasciitis and 825 patients with cellulitis were included. With an Laboratory Risk Indicator for Necrotizing Fasciitis cut-off score ≥6, the sensitivity was 43% (95% confidence interval 34% to 53%), specificity was 83% (95% confidence interval 80% to 86%), positive predictive value was 25% (95% confidence interval 20% to 30%), and negative predictive value was 92% (95% confidence interval 91% to 93%); with an Laboratory Risk Indicator for Necrotizing Fasciitis cut-off score ≥8, the sensitivity was 27% (95% confidence interval 19% to 37%), specificity was 93% (95% confidence interval 91% to 94%), positive predictive value was 33% (95% confidence interval 25% to 42%), and negative predictive value was 91% (95% confidence interval 90% to 92%). The area under the receiver operating characteristic curve for accuracy of the Laboratory Risk Indicator for Necrotizing Fasciitis score was 0.696 (95% CI 0.640 to 0.751).

**Conclusion:**

The Laboratory Risk Indicator for Necrotizing Fasciitis score may not be an accurate tool for necrotizing fasciitis risk stratification and differentiation between severe cellulitis and necrotizing fasciitis in the emergency department setting based on our study.

## Introduction

Necrotizing fasciitis (NF) is a life-threatening soft tissue infection. Globally, the mortality is high even with modern management modalities, ranging from 19% to 30% for all affected NF sites, including the neck, trunk, perineum, and extremities [1–6]. Early diagnosis of NF is critical for carrying out aggressive surgical debridement and decreasing the mortality and morbidity of NF patients. However, it is a clinical challenge to distinguish NF from non-necrotizing soft-tissue infections in the emergency department (ED).

The diagnosis of NF primarily relies on clinical suspicion [7], although many diagnostic adjuncts have been developed to assist with prompt and accurate diagnosis of NF. These diagnostic tools include soft tissue echo [8], enhanced computed tomography (CT) [9], magnetic resonance imaging (MRI) [10], laboratory tests, and scoring systems [11–13]. Plain radiography had poor sensitivity to rule-out NF [14]. CT and MRI can recognize the subtle signs of NF, but may delay the definitive surgical intervention [14]. Abnormal biochemical tests may aid in the diagnosis of NF but are not specific [5,11,15], because these abnormal changes may also be seen in other causes of infection or inflammation. The Laboratory Risk Indicator for Necrotizing Fasciitis (LRINEC) score was developed as a tool for distinguishing NF from other soft tissue infections by Wong et al [13]. It consists of six laboratory tests (see Table 2 in [13]), including white blood cell (WBC) count, hemoglobin, sodium, glucose (≤ 180 and >180 mg/dL), creatinine (≤ 1.6 and >1.6 mg/dL), and C-reactive protein. The maximum score is 13, and a score of ≥6 is suspicious of NF with a probability of 50 to 75%, whereas a score of ≥8 is strongly predictive of NF with a probability of more than 75%.

This score has shown robust performance in an initial retrospective external validation [13]. However, in recent validation studies with retrospective chart reviews, the LRINEC score has had inadequate sensitivity in diagnosing NF, ranging from 36 to 83% [5,11,16–25]. Furthermore, two systemic reviews of the LRINEC score showed opposite results [14,26]. One study concluded that the LRINEC score is a useful clinical tool in the diagnosis of NF, but the other revealed that the LRINEC score had poor sensitivity of 68.2% with an LRINEC score cutoff ≥6 and 40.8% with an LRINEC score cutoff ≥8. To our knowledge, no prospective validation study of the LRINEC score has been conducted.

The LRINEC score varied markedly depending on the affected part of the body, with limbs scoring 6, groin 6.8, and chest/trunk 7.3 [26]. This reflects the fact that patients with NF involving different body parts may have unique characteristics. Since the majority of NF involves the extremities [5,16,19,27,28], the purpose of this study is to prospectively evaluate the performance of the LRINEC score for the diagnosis of patients with NF of the extremities.

## Method

### Study design

This prospective and observational cohort study was conducted in one suburban, academic, tertiary care hospital in Taiwan with 1,300 beds and approximately 80,000 annual ED visits. The study period was from April 2015 to December 2016, and the study protocol was approved by Chang Gung Medical Foundation, Institutional Review Board. The ethics authorization code was 100-4178B. Written informed consent was required for the study protocol.

The study enrolled all patients aged 18 years or older who were admitted to the ward through the ED with an ED diagnosis of cellulitis or NF affecting the extremities. The ED physicians made the clinical diagnosis of cellulitis or NF based on symptoms, signs, laboratory exams, and sometimes imaging exams or consultation opinions if available. Patients were excluded if they were not admitted via the ED, were younger than 18 years old, had previously received antibiotics or debridement, or had lesions involving the neck or trunk area. The following demographic and clinical data were collected from all of the enrolled patients by research assistants: age, gender, vital signs at ED triage, symptoms and signs, mortality, and comorbidities, including diabetes mellitus, liver cirrhosis, cancer, and peripheral vascular disease. Laboratory examinations were ordered at the time of admission to the ED, including WBC count, hemoglobin, creatinine, sodium, glucose, C-reactive protein, blood culture, and wound culture if wound presented or operation performed. Hypotension was defined as systolic blood pressure less than 90 mmHg in ED triage.

The study endpoint was when patients were discharged from the ward. The enrolled patients were divided into two groups, the NF group and the severe cellulitis group, according to the following criteria: The diagnosis of NF was confirmed by the final operative and pathologic findings of NF. The operative findings included presence of necrotic fascia and pus-like fluid. The pathology evidence of NF consisted of necrosis, polymorphonuclear infiltration, vasculitis and thrombosis in the tissue. Severe cellulitis was defined by the administration of parenteral antibiotics for more than 48 hours or the presence of an abscess requiring surgical debridement; the same definition was used in the initial LRINEC developmental paper by Wong et al [13]. Patients who had a length of stay <48 hours or who were administered oral antibiotics were excluded.

### Outcomes and measures

The LRINEC score was calculated for every enrolled patient using six laboratory variables. Since an LRINEC score of ≥6 indicates a 50 to 75% probability of NF, and an LRINEC score of ≥8 indicates a greater than 75% probability [13], the sensitivity, specificity, positive predictive value (PPV), and negative predictive value (NPV) of cut-off scores of 6 and 8 were calculated separately. Receiver operating characteristic (ROC) curves with 95% confidence intervals (CI) was used to evaluate the predictive accuracy of the LRINEC score.

### Statistical analysis

All variables between the NF group and the severe cellulitis group were compared and analyzed using SPSS statistical software (IBM SPSS 24, Armonk, NY: IBM Corp). Univariate analysis was performed to identify the risk factors of necrotizing fasciitis. The chi-squared test or Fisher exact test were used as appropriate for proportions with dichotomous variables, along with the Mann-Whitney U test for medians with numerical variables. A p-value < 0.05 was considered to be significant. ROC curves with 95% CI was used for the predictive accuracy of the LRINEC score. The predictive accuracy of the risk score was expressed as the area under the ROC curve (AUROC).

## Results

**Fig 1:**
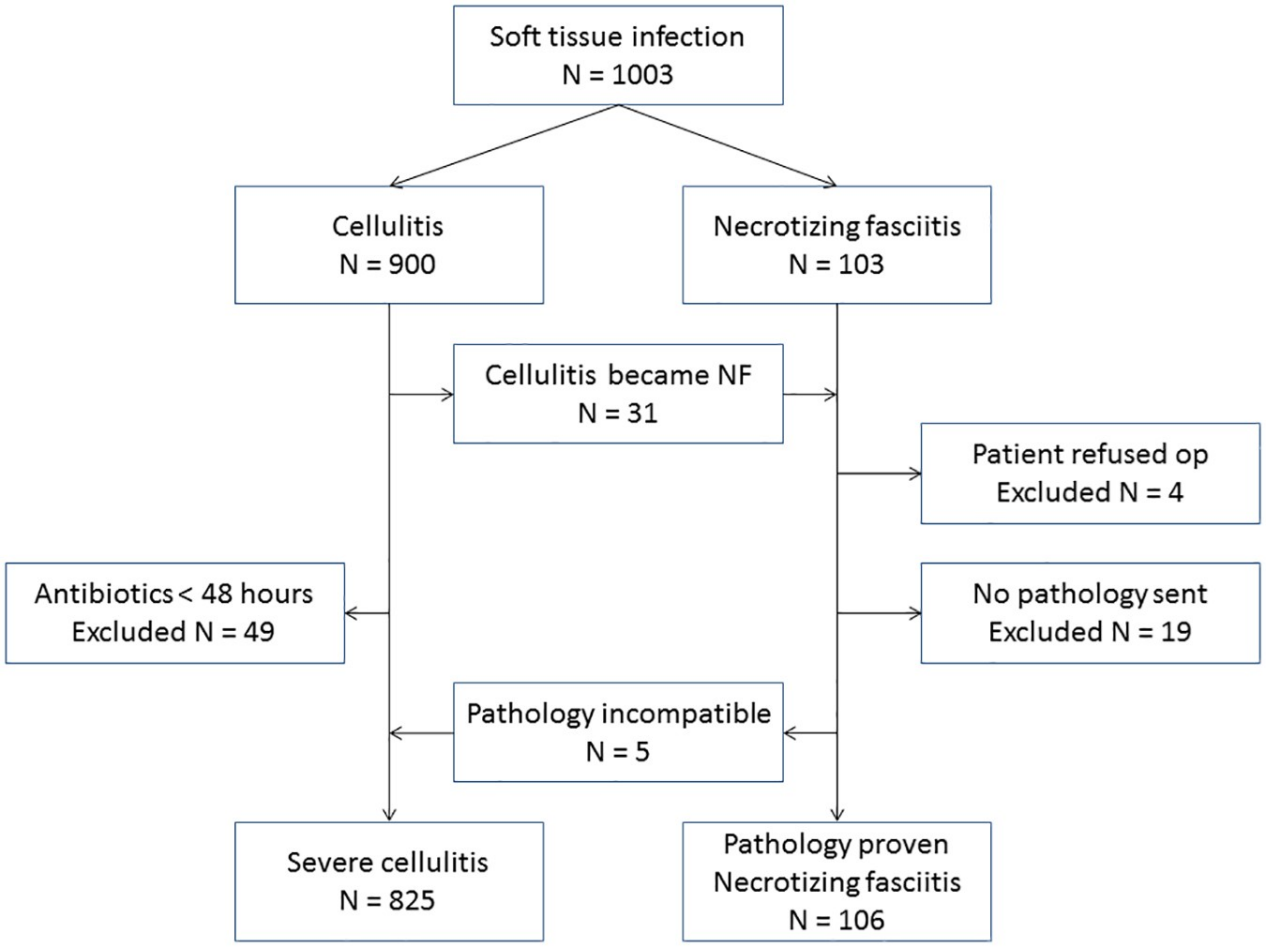
Flow chart of patient inclusion.

A total of 1,003 patients with age greater than or equal to 18 who were admitted to the ward through the ED with an ED diagnosis of cellulitis or NF of the extremities were enrolled (Fig 1). Of these, 900 were initially diagnosed with cellulitis and 103 with NF. Among patients with NF, 23 were excluded for the following reasons: four patients refused surgery and 19 patients underwent surgery, but the pathologic reports were unavailable. Five patients were reclassified as severe cellulitis due to inconsistent pathologic reports for NF, including acute inflammation, subepidermal pustules, and gouty tophi. Therefore, a total of 106 patients had both operative and pathologic reports that were consistent with NF. Among the 900 patients with cellulitis, 31 patients developed NF during ward admission, and 49 patients received intravenous antibiotics for less than 48 hours. In addition, five patients were reclassified as severe cellulitis from NF due to inconsistent pathologic reports. Therefore, a total of 825 patients with cellulitis corresponded to the definition of severe cellulitis.

Table 1 demonstrates the demographic and clinical variables of the severe cellulitis group and NF group. Among the comorbidities, only peripheral vascular disease was significantly more common in the severe cellulitis group than in the NF group (p = 0.015). All variables of the LRINEC score except for hemoglobin were significantly higher in the NF group than in the severe cellulitis group (all p < 0.05).

**Table 1:** Demographic and clinical variables of patients used in the validation cohort.

	Necrotizing fasciitis (n = 106)	Severe cellulitis (n = 825)	p-value
**Age** ^a^	68 (58–78)	68 (56–78)	0.847
**Male** ^b^	70 (66.0)	509 (61.7)	0.386
**Comorbidities** ^b^			
Diabetes mellitus	51 (48.1)	362 (43.9)	0.409
Liver cirrhosis	13 (12.3)	99 (12.0)	0.937
Cancer	12 (11.3)	92 (11.2)	0.958
Peripheral vascular disease	1 (0.9)	63 (7.6)	0.015
**Variables on admission** ^b^			
Temperature > 38.0°C	26 (24.5)	114 (13.8)	0.004
Hypotension^c^	15 (14.2)	14 (1.7)	< 0.001
Pain	100 (94.3)	719 (87.2)	0.032
Hemorrhagic bullae	14 (11.7)	22 (2.7)	< 0.001
**LRINEC variables** ^a^			
C-Reactive Protein, mg/L	122.4 (44.4–206.8)	31.4 (7.9–99.9)	< 0.001
Total white cell count, per mm^3^	13.8 (10.1–18.0)	9.7 (7.2–13.2)	< 0.001
Hemoglobin, g/dL	12.3 (11.0–13.5)	12.2 (10.5–13.5)	0.484
Sodium, mmol/L	135 (132–137)	137 (133–139)	< 0.001
Creatinine, μmol/L	110.5 (75.8–160.9)	87.5 (69.9–118.3)	0.001
Glucose, mmol/L	8.9 (6.7–14.1)	7.8 (6.4–10.8)	0.019
**Mortality rate** ^b^	9 (8.5)	18 (2.2)	< 0.001

^a^ The data given as median with interquartile range in parentheses

^b^ The data given as the number of patients, with the percentage in parentheses

^c^ Hypotension was defined as the systolic blood pressure less than 90 mmHg at ED triage

The LRINEC score was calculated for each patient and summarized according to cut-off scores of 6 and 8 (Table 2 and S1 File). The mean LRINEC score of all patients was 3.3 ± 2.8. The mean LRINEC score was 5.3 ± 3.4 for the NF group and 3.1 ± 2.6 for the severe cellulitis group, and the difference was statistically significant (p < .001).

**Table 2:** LRINEC score with validation cohort.

LRINEC score	Necrotizing fasciitis	Cellulitis	Total
≥ 8	29 (33%)	60 (67%)	89
6–7	17 (18%)	79 (82%)	96
≤ 5	60 (8%)	686 (92%)	746
Total	106 (11%)	825 (89%)	931

The bacterial culture results and average LRINEC score of patients with necrotizing fasciitis was shown in the Table 3. The most common microorganism of monomicrobial necrotizing fasciitis was *Vibrio vulnificus*. Except *Coag(-) staphylococcus* which may be due to contamination, the patients with *Vibrio vulnificus* and *Aeromonas hydrophila* infection had average LRINEC score of 3.9 and 3.5, which were lower than the mean of the whole NF group.

**Table 3:** The bacterial culture results and average LRINEC score of patients with necrotizing fasciitis.

Culture results^a^	Case number	Average LRINEC score^b^
*Vibrio vulnificus*	19	3.9 (0–10)
*Staphylococcus aureus* (ORSA)	12	5.9 (1–11)
*Coag(-) staphylococcus*	6	1.8 (0–3)
*Aeromonas hydrophila*	4	3.5 (1–7)
Other monomicrobial^c^	14	7.0 (2–11)
Polymicrobial	28	6.3 (0–13)
No growth	23	5.1 (0–11)
Total	106	5.3 (0–13)

^a^ The bacterial culture result was positive if the bacteria grew in either blood culture, or wound culture.

^b^ The data given as mean with the range in parentheses.

^c^ Other monomicrobial infections including *β-streptococcus group A* (2 cases), *β-streptococcus group B* (2 cases), *Streptococcus equisimilis* (2 cases), *β-streptococcus group non ABD* (1 case), *Oxacillin-sensitive Staphylococcus aureus* (1 case), *Pseudomonas aeruginosa* (2 cases), *Klebsiella pneumoniae* (1 case), *Escherichia coli* (1 case), *Enterobacter cloacae* (1 case), and *Shewanella putrefaciens* (1 case).

ORSA, *Oxacillin-resistance Staphylococcus aureus*

**Fig 2:**
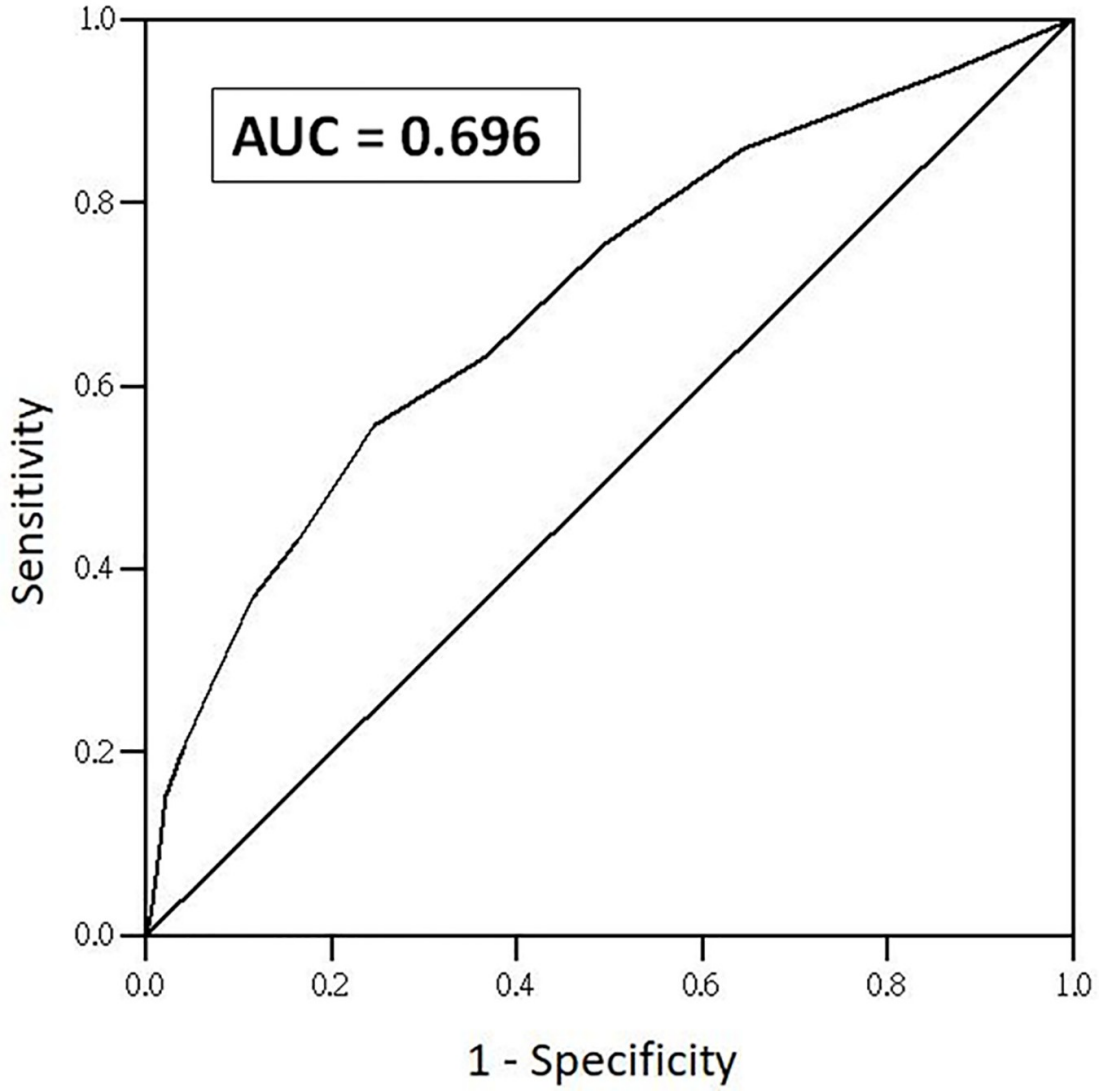
The AUROC for accuracy of the LRINEC score. With an LRINEC cut-off score ≥8, the AUROC for accuracy of the LRINEC score was 0.696.

With an LRINEC cut-off score ≥6, the sensitivity was 43% (95% CI 34% to 53%), specificity was 83% (95% CI 80% to 86%), PPV was 25% (95% CI 20% to 30%), and NPV was 92% (95% CI 91% to 93%); with an LRINEC cut-off score ≥8, the sensitivity was 27% (95% CI 19% to 37%), specificity was 93% (95% CI 91% to 94%), PPV was 33% (95% CI 25% to 42%), and NPV was 91% (95% CI 90% to 92%). The AUROC for accuracy of the LRINEC score was 0.696 (95% CI 0.640 to 0.751) (Fig 2).

## Discussion

To our knowledge, our study is the first prospective cohort study to validate the LRINEC score with a large number of patients. This study revealed that the LRINEC score had low accuracy with an AUROC of 0.696, and a poor sensitivity of 43% with an LRINEC score ≥6 and 27% with an LRINEC score ≥8. Our results suggest that the ability of the LRINEC score to discriminate NF and severe cellulitis in the extremities is inadequate. The LRINEC score should be used with caution as a routine diagnostic tool of NF in the ED.

This prospective validation study showed that the sensitivity of the LRINEC score was poor, which is consistent with the results of previous retrospective and meta-analysis studies, in which the sensitivity ranged from 36 to 83% [5,11,14,16–25]. Using the insensitive LRINEC score to rule out patients with NF may potentially cause delayed diagnosis, delayed surgical management, and increased unfavorable outcomes, because high percentages of patients with NF would be misdiagnosed as having severe cellulitis.

Regarding the accuracy of the LRINEC score, the retrospective validation and meta-analysis studies showed conflicting results. The largest retrospective validation study (NF group: 233, severe cellulitis group: 1394) by Liao et al [22]. showed that the AUROC curve of the LRINEC score for NF was only moderate, 0.779. The author concluded that the LRINEC score alone is not useful for the early recognition of NF. The second largest retrospective validation study (NF group: 47, severe cellulitis group: 948) by Neeki et al [23] revealed that the sensitivity with an LRINEC score ≥6 was 36%, and the specificity was 89%, which are similar to our results (sensitivity 43% and specificity 83%). The greater than 50% of false negative rate in confirmed cases of NF may potentially cause delayed surgical management, and increased mortality. The greater than 10% false positive rate in confirmed cases of severe cellulitis may lead to unnecessary clinical workups with laboratory or imaging studies for NF, which further exacerbates the clinical load of the ED and the financial burden of patients. One recent meta-analysis also found that an LRINEC score ≥6 was associated with a sensitivity of 68.2% and specificity of 84.8% [14].

However, some studies have reported that the LRINEC score is a useful clinical tool with good performance for the diagnosis of NF [16,26]. One report retrospectively validated the LRINEC scores with an AUROC of 0.925, sensitivity of 76.3%, specificity of 93.1%, and positive and negative predictive values of 95.5% and 88.1% based on an LRINEC value of ≥6 to distinguish NF from severe cellulitis [16]. The conclusion was that the LRINEC score is a useful and robust scoring system as an adjunct for the early diagnosis of NF. One meta-analysis included 16 studies with 846 patients, and an AUROC of 0.925 was obtained [26]. The study revealed that the LRINEC score is a useful clinical determinant in the diagnosis of NF. Despite the paradoxical conclusions from these retrospective validation and meta-analysis studies, our prospective validation results indicated that accuracy of the LRINEC score was poor, and the LRINEC may not be an accurate tool for NF risk stratification and differentiation between severe cellulitis and NF in the ED setting based on our study.

The reasons why our results were different from those of the LRINEC developmental study may be due to several factors. First, the patient characteristics of the study cohorts were different. The patients in our study were older (median age 68 versus 56), exclusively from the ED, and had higher percentage of diabetes mellitus, peripheral vascular disease, and fever, compared to patients in the study of Wong et al. Second, only patients with NF involving the limbs were included in our study, which might have biased the validation results. As previously mentioned, the LRINEC score of NF varied markedly depending on the affected body part, and the limbs had the lowest score of 6, compared to our average LINREC score of 5.3[26]. Finally, the most common microorganism of monomicrobial necrotizing fasciitis in Europe and Singapore was *Group A Streptococcus* [5,11,20]. However, the most common microorganism of monomicrobial necrotizing fasciitis in our study was *Vibrio vulnificus*. In our study, the patients with *Vibrio vulnificus* and *Aeromonas hydrophila* infection had lower LRINEC score than the patients with other bacteria. The different causative microorganisms have different pathogeneses and clinical courses, which may lead to different LRINEC score and may have biased the results of the validation.

Our study showed that the LRINEC score failed to classify patients with severe soft tissue infection in the extremities into low-, moderate-, and high-risk groups of necrotizing fasciitis. This raises a basic question: Is clinical judgement better than the LRINEC score in the diagnostic process of NF? A suggested clinical pathway to manage soft tissue infection was proposed in the LRINEC developmental study [13]. However, the first step of the clinical pathway is based on clinical evaluation and thorough physical examinations to identify NF. Then, applying the LRINEC score stratifies equivocal cases into low, intermediate, and high-risk groups. In this clinical pathway, the value of clinical judgement seems to be superior to the LRINEC score. Since NF is a clinical diagnosis that relies primarily on clinical judgement, the LRINEC score seems to have a less important role in the diagnostic process. In our study, the ED physicians made the clinical diagnosis of NF primarily based on the combination of symptoms, signs, laboratory exams. In regard to doubtful cases of NF, imaging exams, including ultrasound or computed tomography, may be performed as auxiliary diagnostic tool. Then, the final diagnosis was achieved through the discussion between orthopedic surgeons and ED physicians. One previous study attempted to modify the LRINEC score by adding clinical parameters and different laboratory parameters in order to improve the score with a higher PPV without losing specificity [11]. However, the scoring system should not be regarded as an independent method of diagnosis. Clinical judgement and diagnosis should be based on the combination of age, gender, comorbidities, physical examinations, laboratory studies, and imaging. Comparing the LRINEC score derived from logistic regression, the algorithms of machine learning, such as decision trees or neural network, may help emergency physicians to develop clinical decision rules and compute predictive results more rapidly. Further research is recommended to develop new clinical decision rules with high accuracy, convenience, and validation, as well as a multicenter prospective comparative studies comparing such clinical decision rules with the LRINEC score and other diagnostic tools.

## Limitations

There were some limitations in our prospective study. First, we lacked gold standard test to define all cases of the soft tissue infection. The diagnosis of NF can be confirmed by the final operative and pathologic findings of NF. However, the diagnosis of severe cellulitis can only be confirmed by clinical course. We did not design all enrolled cases to undergo CT or MRI imaging, or percutaneous biopsy because none of these diagnostic tests can be the gold standard test of NF and used for the comparison of accuracy [7]. Second, only patients with NF involving the limbs were included in our study. Future prospective validation studies are warranted to determine whether the LRINEC score is a useful tool for discriminating NF from severe cellulitis in other parts of the body. Finally, this was a single-hospital study, and the patient characteristics of the study cohort may be different from other institutions. Thus, our findings may not be applicable to other institutions with different patient characteristics, hospital features, and levels of care. In order to further test the validity of the LRINEC score, a multi-center study from different countries and with a larger sample should be conducted in the future.

## Conclusions

This study has shown that the LRINEC score may not be an accurate tool for NF risk stratification and differentiation between severe cellulitis and NF in the ED setting. The LRINEC score should be used with caution as a routine diagnostic tool of NF in the ED. A multicenter prospective comparative studies comparing the LRINEC score with other diagnostic tools may be needed in the future.

## Supporting information

S1 File Anonymized data for the LRINEC score calculation. (XLSX)
