# Correction: Prospective Validation of the Laboratory Risk Indicator for Necrotizing Fasciitis (LRINEC) Score for Necrotizing Fasciitis of the Extremities

**DOI:** 10.1371/journal.pone.0270726

**Published:** 2022-06-24

**Authors:** Cheng-Ting Hsiao, Chia-Peng Chang, Tsung-Yu Huang, Yi-Chuan Chen, Wen-Chih Fann

## Notice of republication

This article [1] was republished on June 15, 2022 to remove Table 1. Table 1 was adapted from Table 2 in article [2], published in 2004 by Wolters Kluwer, which is not offered under a CC-BY license. Please download this article again to view the correct version. Article [2] is cited as reference 13 in article [1].

The sentence in the Introduction referencing Table 1 has therefore been updated to “It consists of six laboratory tests (see Table 2 in [13]), including white blood cell (WBC) count, hemoglobin, sodium, glucose (≤180 and >180 mg/dL), creatinine (≤1.6 and >1.6 mg/dL), and C-reactive protein. The maximum score is 13, and a score of ≥6 is suspicious of NF with a probability of 50 to 75%, whereas a score of ≥8 is strongly predictive of NF with a probability of more than 75%.”
